# Utility of Combining Prostate Health Index and Magnetic Resonance Imaging for the Diagnosis of Prostate Cancer

**DOI:** 10.1111/iju.70024

**Published:** 2025-03-17

**Authors:** Yoshitaka Sekine, Yuji Fujizuka, Shun Nakazawa, Yusuke Tsuji, Akira Ohtsu, Yoshiyuki Miyazawa, Seiji Arai, Masashi Nomura, Hidekazu Koike, Hiroshi Matsui, Kei Shibuya, Kazuto Ito, Hayato Ikota, Kazuhiro Suzuki

**Affiliations:** ^1^ Department of Urology Gunma University Graduate School of Medicine Maebashi Japan; ^2^ Department of Radiation Oncology Gunma University Graduate School of Medicine Maebashi Japan; ^3^ Department of Urology Kurosawa Hospital Takasaki Japan; ^4^ Department of Diagnostic Pathology Gunma University Hospital Maebashi Japan

**Keywords:** PI‐RADS, prostate biopsy, prostate cancer, prostate health index, PSA

## Abstract

**Objective:**

In this study, we compared the prostate health index (PHI) and the Prostate Imaging Reporting and Data System (PI‐RADS) before prostate biopsy and evaluated the utility of combining them in the diagnosis of prostate cancer (PC).

**Methods:**

Between November 2021 and March 2023, 105 patients admitted to Gunma University Hospital for prostate biopsy after undergoing both MRI and PHI measurements were enrolled in this study. We investigated the diagnostic impacts of [−2]proPSA related indexes on clinically significant PC (csPC) and the complementary effects of PHI and PI‐RADS.

**Results:**

The median of PHI was 59.9, and 72 patients (69%) were diagnosed with PC. The receiver operating characteristic (ROC) curve for patients diagnosed with PC indicated an area under the curve (AUC) of 0.816 for PHI and 0.753 for PI‐RADS. For PHI, when the sensitivity was 90% and 95%, the specificity was 51.5% and 45.5%, respectively. Both are higher than those of the conventional PSA‐related indices. In addition, the ROC curve in patients with a diagnosis of csPC (*n* = 67) indicated an AUC of 0.793 for PHI and 0.746 for PI‐RADS. Furthermore, if biopsy was restricted to patients with PI‐RADS ≥ 4 or PHI ≥ 38.1, 21% of unnecessary biopsies could be avoided, with only one (1.5%) patient with csPC being missed.

**Conclusions:**

PHI is more available for detecting csPC than PSA and PSA F/T. Moreover, there is a possibility that unnecessary prostate biopsies can be avoided by combining PHI and PI‐RADS.

**Abbreviations**
AUC: area under the curve
csPC: clinically significant prostate cancer
DRE: digital rectal examination
F/T: free to total ratio
FPR: false positive rate
mpMRI: multiparametric magnetic resonance imaging
MRI: magnetic resonance imaging
PC: prostate cancer
PHI: prostate health index
PI‐RADS: Prostate Imaging Reporting and Data System
PSA: prostate specific antigen
ROC: receiver operating characteristics
TRUS: transrectal ultrasound

## 1 Introduction

Prostate cancer (PC) is one of the most common types of cancer in men [1]. Although prostate‐specific antigen (PSA) is a useful tumor marker for PC detection, its low specificity and the detection of clinically insignificant cancers are problematic [2]. The Prostate Health Index (PHI) is calculated using the formula (−2)proPSA/free PSA × √total PSA, and has been reported to be more useful in detecting PC and clinically significant PC (csPC) than the existing markers PSA and PSA free to total ratio (F/T) [3, 4]. It was approved by the FDA in the United States in 2012 [5]. Meanwhile, a study conducted in Japan involving 421 men aged 50–79 years, with PSA levels above age‐specific cutoffs and below 10 ng/mL, demonstrated that PHI was superior to PSA and PSA F/T in diagnosing PC and detecting csPC [6]. The PHI was then covered by insurance in 2021 and can now be used in clinical practice.

It has also been reported that magnetic resonance imaging (MRI) is useful for PC detection. The Prostate Imaging Reporting and Data System version 2 (PI‐RADS v2) is used to evaluate the prostate [7] and targeted biopsy is recommended for lesions with a PI‐RADS of 3 or higher [8].

Few studies in Japan have examined the PHI; therefore, we first examined its usefulness in detecting PC in clinical practice. We also investigated the possibility of avoiding unnecessary biopsies in the Japanese population without compromising the ability to detect csPC by combining it with MRI.

## 2 Methods

### 2.1 Patients and Study Design

This was a prospective, observational study. Between November 2021 and March 2023, 105 patients admitted to Gunma University Hospital for prostate biopsy were enrolled. Study inclusion criteria were (1) serum PSA higher than the age‐stratified PSA cutoffs of 3.0 ng/mL at ages 50 to 64 years, 3.5 ng/mL at 65–69 years, and 4.0 ng/mL at 70 years or older, and 10 ng/mL or less; (2) measurement of PHI before prostate biopsy; and (3) MRI before prostate biopsy. The exclusion criteria were a history of surgery for benign prostatic hyperplasia, bacterial urinary infection before biopsy, and the use of steroidal or nonsteroidal anti‐androgens or 5‐alpha reductase inhibitors within 3 months of study start. The urologist performed a digital rectal examination (DRE) and transrectal ultrasound (TRUS) before prostate biopsy. Prostate volumes were calculated from TRUS images.

### 2.2 MRI Protocol

All patients underwent multiparametric MRI (mpMRI) before prostate biopsy using a 1.5 or 3T device without an endorectal coil. Diffusion‐weighted, T2W, and dynamic contrast‐enhanced imaging were evaluated and scored by a radiologist using PI‐RADS v2.1 before the biopsy. A PI‐RADS of 3 or higher was defined as a positive finding.

### 2.3 Biopsy Protocol

Perineal prostate biopsy was performed under lumbar or general anesthesia by urologists who had no specific experience in reading MRI. Systematic biopsy was performed at 8–20 cores by the age‐adjusted and prostate volume‐adjusted prostate biopsy method as previously reported [9]. Cognitive targeted biopsy was done for patients with suspicious lesions (PI‐RADS ≥ 3) after systematic biopsy. Two samples were collected from each of the target lesions. The biopsy samples were evaluated by pathologists using the Gleason Grade Group [10].

### 2.4 Statistical Analysis

In this study, we defined non‐csPC as 1–2 positive biopsy cores and a Gleason Grade Group of 1. The Mann–Whitney *U* test was used for continuous variables (age, PSA, PSA F/T, %[−2]proPSA, [−2]proPSA/%f‐PSA, and PHI). Independence was estimated using the chi‐squared test for categorical variables (MRI, DRE, and TRUS). The predictive accuracy of PC or csPC, PSA, PSA F/T, PHI, %[−2]proPSA, (−2)proPSA/%f‐PSA, and PI‐RADS score was evaluated using the area under the curve (AUC) of the receiver operating characteristic (ROC) analysis. *P*‐values ≤ 0.05 were considered statistically significant. SPSS Statistics Ver. 25 (IBM Corp. IL, USA) was used for the statistical analyses.

### 2.5 Institutional Ethics Approval

This study was approved by the Institutional Review Board of the Gunma University Hospital (approval no. 2022‐007, 1991).

## 3 Results

### 3.1 Patient Characteristics

Table 1 shows the clinical characteristics of the 105 patients. The mean age, PSA level, and PHI were 68.4 years, 6.19 ng/mL, and 68.9, respectively. There were 4 (3.8%) patients with PHI of 0–27.1, 11 (10.4) patients with PHI of 27.2–36.0, 32 (30.4) patients with PHI of 36.1–55.0, and 58 (55.2%) patients with PHI over 55.1. PC was found in 69% (*n* = 72) using prostate biopsy. The median age, PSA, PSA F/T, %[−2]proPSA, (−2)proPSA/%f‐PSA, and PHI levels were significantly different between PC and non‐PC groups. Regarding MRI, 93% of patients had PI‐RADS ≥ 3 lesions with PC, while 72.7% of patients had PI‐RADS ≥ 3 lesions without PC (*p* = 0.004). In contrast, no significant differences were observed between the PC and non‐PC groups in DRE (*p* = 0.38) and TURS (*p* = 0.92).

**TABLE 1:** Patient clinical background.

Variable	Total (*n* = 105)	PC (*n* = 72)	non‐PC (*n* = 33)	*p*
Age (years)	68.4 ± 9.0 (30–86)	70.4 ± 8.1 (51–86)	64.0 ± 9.5 (30–76)	< 0.001
PSA (ng/mL)	6.19 ± 1.73 (3.11–9.58)	6.43 ± 1.70 (3.46–9.58)	5.67 ± 1.68 (3.11–9.06)	0.027
PSA F/T	0.15 ± 0.07 (0.05–0.43)	0.14 ± 0.06 (0.05–0.43)	0.18 ± 0.07 (0.05–0.36)	0.003
%[−2]proPSA	2.80 ± 1.51 (0.76–9.50)	3.17 ± 1.58 (0.88–9.50)	2.01 ± 1.00 (0.76–5.96)	< 0.001
[−2]proPSA/%f‐PSA	172.70 ± 104.88 (35.99–662.33)	201.96 ± 110.31 (35.99–662.33)	108.87 ± 51.21 (57.17–246.68)	< 0.001
PHI	68.90 ± 38.69 (17.80–230.50)	79.23 ± 40.50 (17.80–230.50)	46.36 ± 21.51 (22.10–121.20)	< 0.001
PI‐RADS				
1/2	14 (13.3)	5 (7.0)	9 (27.3)	
3	18 (17.1)	8 (11.1)	10 (30.3)	
4	46 (43.8)	34 (47.2)	12 (36.3)	
5	27 (23.8)	25 (34.7)	2 (6.1)	
MRI (P/N)	91 (86.7)/14 (13.3)	67 (93.0)/5 (7.0)	24 (72.7)/9 (27.3)	0.004
DRE (P/N)	14 (13.3)/91 (86.7)	11 (15.2)/61 (84.7)	3 (9.0)/30 (91.0)	0.38
TRUS (P/N)	28 (26.7)/77 (73.3)	19 (26.4)/53 (73.6)	9 (37.5)/24 (62.5)	0.92

*Note:* Values are expressed as mean ± standard deviation (min to max) or absolute frequency (percentage).

Abbreviations: DRE, digital rectal examination; F/T, free/total; MRI, magnetic resonance imaging; N, negative; P, positive; PHI, prostate health index; PI‐RADS, Prostate Imaging Reporting and Data System; PSA, prostate specific antigen; TRUS, transrectal ultrasound.

### 3.2 Prediction of PC and csPC

**FIGURE 1:**
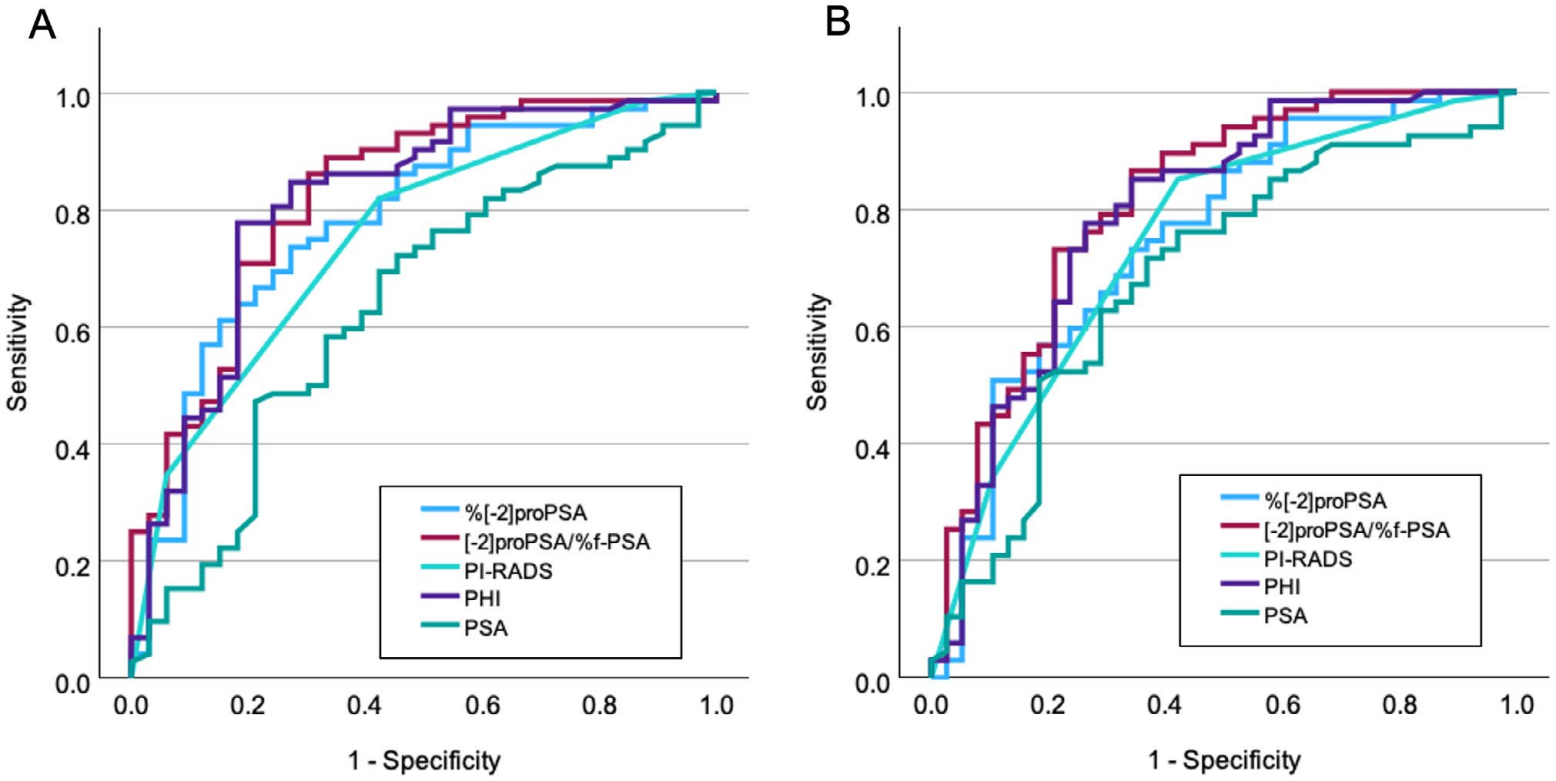
ROC curves for PHI, PI‐RADS, PSA, %[−2]proPSA, and (−2) proPSA/% f‐PSA for predicting PC (A) and csPC (B). csPC, clinically significant prostate cancer ; PC, prostate cancer; PHI, prostate health index; PI‐RADS, Prostate Imaging Reporting and Data System; PSA, prostate‐specific antigen; ROC, receiver operating characteristic.

Table 2 and Figure 1A show AUC‐ROC and false positive rate (FPR) at 90% sensitivity for PSA, PSA F/T, PHI, %[−2]proPSA, (−2)proPSA/%f‐PSA, and PI‐RADS for PC detection. The ROC curve for patients diagnosed with PC showed an AUC of 0.816 for the PHI and 0.753 for the PI‐RADS. For PHI, when the sensitivity was 90% and 95%, the specificity was 51.5% and 45.5%, and the cutoffs of PHI were 43.5 and 38.1, respectively. The specificities were higher than those of PSA and PSA F/T. The AUC of PHI was higher than those of PSA, PSA F/T, and PI‐RADS. In addition, the ROC curve for patients diagnosed with csPC (*n* = 67) showed an AUC of 0.793 for PHI and 0.746 for PI‐RADS (Figure 1B). The AUC of PHI was higher than those of PSA, PSA F/T, and PI‐RADS. For PHI, when the sensitivity was 90% and 95%, the specificity was 47.4% and 42.1%, and the cutoffs of PHI were 43.5 and 39.7, respectively. The specificities were also higher than those of PSA and PSA F/T. For (−2)proPSA/%f‐PSA, the AUC was higher than that of PHI for both PC and csPC. Next, we evaluated whether the PHI had a complementary effect on the PI‐RADS. In the PI‐RADS 3 group, the AUC for the diagnosis of PC and csPC was both higher for PHI than for PSA or PSA F/T. (Table S1). Similar results were obtained in the PI‐RADS 1–2 group. (Table S2).

**TABLE 2:** AUC‐ROC and FPR at 90% sensitivity.

Index	AUC‐ROC (95% CI)	FPR at 90% sensitivity
non‐PC vs. PC		
PSA	0.634 (0.518–0.751)	0.848
PSA F/T	0.681 (0.567–0.794)	0.736
PHI	0.816 (0.723–0.910)	0.485
%[−2]proPSA	0.782 (0.686–0.879)	0.545
[−2]proPSA/%f‐PSA	0.827 (0.739–0.914)	0.394
PI‐RADS	0.753 (0.654–0.852)	—
non‐PC + non‐csPC vs. csPC		
PSA	0.684 (0.576–0.793)	0.684
PSA F/T	0.725 (0.621–0.829)	0.579
PHI	0.793 (0.697–0.888)	0.526
%[−2]proPSA	0.751 (0.651–0.852)	0.579
[−2]proPSA/%f‐PSA	0.814 (0.725–0.903)	0.447
PI‐RADS	0.746 (0.645–0.846)	—

Abbreviations: AUC, area under the curve; CI, confidence interval; cs, clinically significant; F/T, free/total; FPR, false‐positive rate; PC, prostate cancer; PHI, prostate health index; PI‐RADS, Prostate Imaging Reporting and Data System; PSA, prostate specific antigen; ROC, receiver operating characteristic.

### 3.3 Examination of Prostate Biopsy Conditions Using the PHI and PI‐RADS Score

**FIGURE 2:**
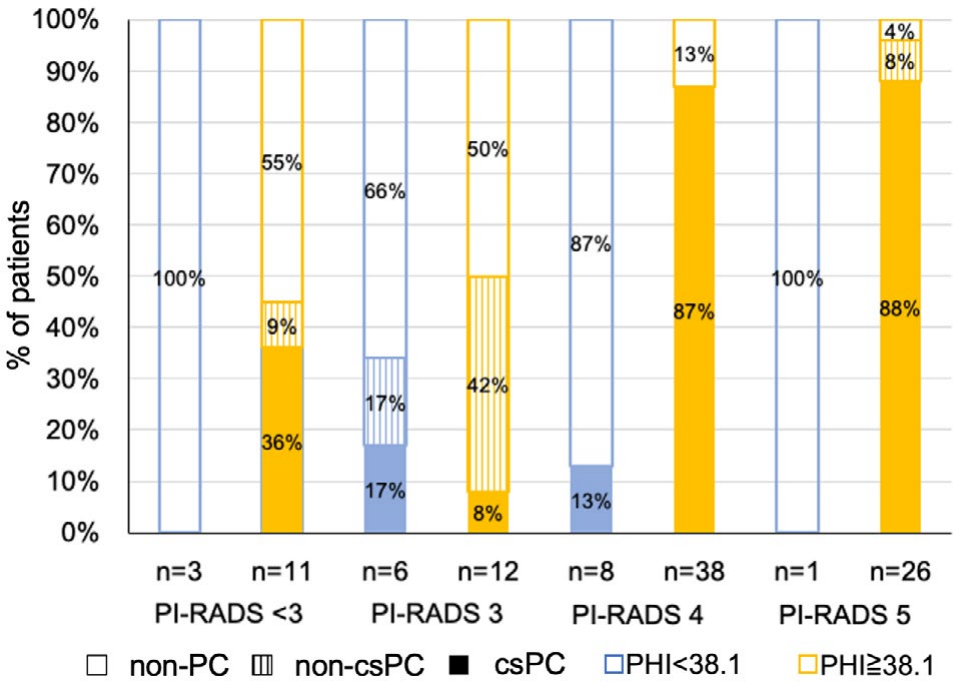
Distribution of clinical results subcategorized by PHI and PI‐RADS. csPC, clinically significant prostate cancer; *n*, number; PC, prostate cancer; PHI, prostate health index; PI‐RADS, Prostate Imaging Reporting and Data System.

Figure 2 shows the pathology results categorized using the PHI and PI‐RADS. If biopsy was restricted to patients with PI‐RADS ≥ 4 or PHI ≥ 38.1, which had a cutoff of 95% sensitivity, 21.1% (the specificity; 0.211) of unnecessary biopsies for non‐PC groups could be avoided with only one patient with csPC being missed. Furthermore, if biopsy was restricted to patients with a PI‐RADS of 5 or PHI ≥ 38.1, 39.5% (the specificity; 0.395) of unnecessary biopsies for non‐PC groups could be avoided, although two cases of csPC would be missed.

## 4 Discussion

The PHI has been used in health insurance‐covered medical treatment in Japan since 2021, and as in the PROPHET study [6], it has been shown to be more useful in clinical practice in Japan than PSA and PSA F/T for detecting both PC and csPC. Furthermore, this is the first report from Japan to show that PHI and mpMRI play a complementary roles in detecting csPC and reducing unnecessary biopsies.

PHI is an auxiliary diagnostic marker for PC and is calculated as (−2)proPSA/free PSA × √total PSA. Several reports have shown that PHI is useful for detecting PC. In the United States, 892 patients with PSA levels between 2 and 10 ng/mL were compared. PHI demonstrated a significantly higher diagnostic capability for PC compared to PSA and PSA F/T. The specificity, when the cutoff value for the 95% sensitivity of cancer diagnosis was used, was also higher at 16.0% compared to PSA (6.5%) and PSA F/T (8.4%) [3]. Furthermore, a systematic review of 65 studies showed that PHI had a high diagnostic accuracy for detecting clinically significant cancer [11]. In Japan, we reported that PHI showed significantly greater specificity for PC as compared with PSA F/T in men with total PSA in the 2.0–10.0 ng/mL range [12]. Moreover, a study was conducted on 421 men aged 50–79 years with PSA levels higher than the age‐stratified PSA cutoffs of 3.0 ng/mL at ages 50–64 years, 3.5 ng/mL at 65–69 years, and 4.0 ng/mL at 70 years or older, and 10 ng/mL or less. In terms of the ROC‐AUC for diagnosing PC, the PHI was 0.767, which was significantly superior to PSA (0.579) and PSA F/T (0.640). Concerning the detection of csPC (Gleason Grade Group 2–5 cancer or cancer involving more than two positive biopsy cores), PHI (0.788) was superior to PSA (0.582) and PSA F/T (0.638) [6]. As a result of this report, PHI has been covered by insurance in Japan and has been available as a diagnostic marker for PC since 2021. This study also demonstrated that the PHI is superior to the existing markers, PSA and PSA F/T, for detecting csPC.

Our results also suggest that the PHI and mpMRI may have complementary roles in detecting clinically significant cancers. Previous reports have shown that in repeat prostate biopsies, the combination of PHI was superior to PI‐RADS alone in detecting csPC [13], and that in Asians, the combination of the two was superior to either PHI or PI‐RADS alone in detecting csPC [14]. In this study, although the number of patients was small, the AUC of PHI was higher than that of PSA and PSA F/T in detecting csPC, even in cases with PI‐RADS scores of ≤ 3, suggesting the usefulness of using PHI even in cases where MRI showed little evidence of PC.

Furthermore, the combined use of the PHI and mpMRI may prevent unnecessary biopsies. In recent years, some studies have investigated combinations of the PHI and PI‐RADS cutoff values. When prostate biopsy is restricted in patients with PI‐RADS 5 or PI‐RADS 3–4 and PHI ≥ 30, 4.2% of clinically significant cancers will be missed, but approximately 50% of unnecessary biopsies can be avoided [14]. Other reports have shown that when biopsy is performed in patients with PI‐RADS 3 and PHI ≥ 43.5, 2.2% of clinically significant cancers will be missed, but approximately 42% of unnecessary biopsies can be avoided [15]. Regarding the cutoff value of PHI, the above reports set the sensitivity at approximately 90%, but in our study, the biopsy positivity rate was high at 70%, so in order to further reduce the oversight of cancer, the cutoff value of PHI was set at 38.1, which corresponds to a sensitivity level of 95%. Our results showed that setting biopsy criteria as PHI ≥ 38.1 or PI‐RADS ≥ 4 would miss one case (1.5%) of clinically significant cancer but would avoid unnecessary biopsies in 21.1% of cases, whereas setting biopsy criteria as PHI ≥ 38.1 or PI‐RADS ≥ 5 would miss two cases (3%) of clinically significant cancer but would avoid unnecessary biopsies in 39.5% of cases. Regarding cases with PI‐RADS 4, although it has been reported that the false positive rate is 29%, and cases with lesion size ≤ 6 mm or previous negative biopsy results may not indicate cancer [16], prostate biopsy is generally recommended. Therefore, if the results of this study are to be used in clinical practice, it is likely that PHI ≥ 38.1 or PI‐RADS ≥ 4 may be one of the criteria for prostate biopsy to avoid unnecessary biopsies.

In addition to the PHI, the PHI density (PHID) has been reported to be useful for detecting csPC. It was reported that the AUC for PHID was 0.835 versus 0.801 for PHI, indicating that PHID had an advantage in comparison with PHI alone to detect any PC, but PHI and PHID performed equally in detecting csPC [17]. Concerning the combination of PHID and PI‐RADS, having a PI‐RADS score ≥ 3 or, if the PI‐RADS score ≤ 2, a PHID ≥ 0.44 was 100% sensitive for csPC [18]. We also investigated PHID but found that it did not outperform PHI in terms of detecting PC or csPC (data not shown).

This study has several limitations. First, many of the patients had high PHI values, and the cutoff values for 90% and 95% sensitivity were higher than in a previous Japanese report (PROPHET study) [6]. In the POPHET study, the median PHI was 37.0, with an interquartile range of 27.7–47.9. At our hospital, there is a possibility that “selection bias,” where higher‐risk cases are selected during the referral process, has contributed to the tendency for higher PHI distribution. In central hospitals, such as university hospitals, where higher‐risk cases are more likely to be referred, it is important to explore a diagnostic flow combining PI‐RADS and PHI that minimizes the risk of missing csPC while avoiding unnecessary biopsies. This approach should not be constrained by the PHI cutoff value of 27.2 for 90% diagnostic sensitivity. This study is a pilot study, and the sample size is small for the analysis. Therefore, further investigations with a larger number of cases are necessary to determine the cutoff values for PHI when combined with MRI. Second, although the prostate biopsies we performed were cognitive biopsies, to evaluate the ability to detect PC more accurately, MRI fusion biopsies are necessary. Moreover, 2 cores per suspicious lesions may not be sufficient and representative, especially without MRI fusion. Third, we did not consider whether 1.5T or 3T MRI was used; it is assumed that the analysis with the same T MRI would give better results because of the possible influence on PI‐RADS between 1.5T and 3T.

In conclusion, the PHI is more suitable for detecting PC than PSA and PSA F/T. Moreover, unnecessary prostate biopsies may be avoided by combining the PHI and PI‐RADS. As an extension of this study, it appears feasible to develop a nomogram or another predictive tool that can assist clinicians in estimating the probability of prostate cancer positivity. This tool would enable the calculation of an individual patient's likelihood of prostate cancer positivity by incorporating various clinical parameters, such as PSA level, PI‐RADS, prostate size, age, PHI, and [−2]proPSA/%f‐PSA, which was better than PHI in terms of AUC –ROC for patients diagnosed with csPC.

## Author Contributions

**Yoshitaka Sekine:** conceptualization, methodology, formal analysis, writing – original draft, writing – review and editing. **Yuji Fujizuka:** data curation. **Shun Nakazawa:** data curation. **Yusuke Tsuji:** data curation. **Akira Ohtsu:** data curation. **Yoshiyuki Miyazawa:** data curation. **Seiji Arai:** data curation. **Masashi Nomura:** data curation. **Hidekazu Koike:** data curation. **Hiroshi Matsui:** data curation. **Kei Shibuya:** data curation. **Kazuto Ito:** supervision. **Hayato Ikota:** data curation. **Kazuhiro Suzuki:** supervision.

## Disclosure

Kazuhiro Suzuki is an Editorial Board member of the International Journal of Urology and a co‐author of this article.

## Ethics Statement

This study was approved by the Institutional Review Board of the Gunma University Hospital (approval no. 2022‐007, 1991).

## Consent

An opt‐out approach was used to obtain informed consent from patients.

## Conflicts of Interest

The authors declare no conflicts of interest.

## Supporting information


Table S1.

Table S2.
