# Supplementary material for: Utility of Combining Prostate Health Index and Magnetic Resonance Imaging for the Diagnosis of Prostate Cancer
Source: Int J Urol. 2025 Mar 17;32(6):658–63. doi: 10.1111/iju.70024 (PMC12146247; doi:10.1111/iju.70024)
Supplement: Supplementary file 1 — Table S1. [file IJU-32-658-s002.docx]

Supplemental Table 1. AUC-ROC and FPR at 90% sensitivity with PI-RADS 3 lesions

| Index | AUC-ROC (95% CI) |
| --- | --- |
| No PC (n=10) vs PC (n=8) |  |
| PSA | 0.450 (0.151-0.749) |
| PSA F/T | 0.688 (0.431-0.944) |
| PHI | 0.700 (0.433-0.967) |
| [-2]proPSA/%f-PSA | 0.699 (0.423-0.952) |
| No PC + non-cs PC (n=12)  vs cs PC (n=6) |  |
| PSA | 0.667 (0.388-0.946) |
| PSA F/T | 0.764 (0.525-1.000) |
| PHI | 0.819 (0.609-1.000) |
| [-2]proPSA/%f-PSA | 0.819 (0.619-1.000) |

AUC; area under the curve, ROC; receiver operating characteristic, FPR; false-positive rate, CI; confidence interval, PSA; prostate specific antigen, F/T; free/total, PHI; prostate health index, PI-RADS; Prostate Imaging Reporting and Data System, Pca; prostate cancer, cs; clinically significant

^a^p-value vs PSA
