# Supplementary material for: Utility of Combining Prostate Health Index and Magnetic Resonance Imaging for the Diagnosis of Prostate Cancer
Source: Int J Urol. 2025 Mar 17;32(6):658–63. doi: 10.1111/iju.70024 (PMC12146247; doi:10.1111/iju.70024)
Supplement: Supplementary file 2 — Table S2. [file IJU-32-658-s001.docx]

Supplemental Table 2. AUC-ROC and FPR at 90% sensitivity with PI-RADS 1-2 lesions

| Index | AUC-ROC (95% CI) |
| --- | --- |
| No PC (n=5) vs PC (n=9) |  |
| PSA | 0.556 (0.226-0.885) |
| PSA F/T | 0.578 (0.276-0.880) |
| PHI | 0.844 (0.629-1.000) |
| [-2]proPSA/%f-PSA | 0.800 (0.564-1.000) |
| No PC + non-cs PC (n=10) vs cs PC (n=4) |  |
| PSA | 0.550 (0.170-0.930) |
| PSA F/T | 0.650 (0.367-0.933) |
| PHI | 0.725 (0.433-1.000) |
| [-2]proPSA/%f-PSA | 0.675 (0.384-0.966) |

AUC; area under the curve, ROC; receiver operating characteristic, FPR; false-positive rate, CI; confidence interval, PSA; prostate specific antigen, F/T; free/total, PHI; prostate health index, PI-RADS; Prostate Imaging Reporting and Data System, Pca; prostate cancer, cs; clinically significant

^a^p-value vs PSA
